# Energetically exploiting lignocellulose-rich residues in anaerobic digestion technologies: from bioreactors to proteogenomics

**DOI:** 10.1186/s13068-023-02432-x

**Published:** 2023-11-28

**Authors:** Jan Struckmann Poulsen, Williane Vieira Macêdo, Torben Bonde, Jeppe Lund Nielsen

**Affiliations:** 1 Department of Chemistry and Bioscience, Aalborg University, Fredrik Bajers Vej 7H, 9220 Aalborg E, Denmark; 2 Department of Biological and Chemical Engineering, Aarhus University, Gustav Wieds Vej, 10 D, 8000 Aarhus C, Denmark; 3 Biofuel Technology A/S, Bredkær Parkvej 58, 8250 Egå, Denmark

**Keywords:** Lignocellulose, Anaerobic digestion, Protein stable isotope probing, Metaproteomics

## Abstract

The biogas produced through anaerobic digestion (AD) of renewable feedstocks is one of the promising alternatives to replace fossil-derived energy. Even though lignocellulosic biomass is the most abundant biomass on earth, only a small fraction is being used towards resources recovery, leaving a great potential unexploited. In this study, the combination of state-of-art genomic techniques and engineered systems were used to further advance the knowledge on biogas production from lignocellulosic-rich residues and the microbiome involved in the anaerobic digestion hereof. A long-term adapted anaerobic microbiome capable of degrading wheat straw as the sole substrate was investigated using protein stable isotope probing (protein-SIP). The results indicated that a diverse microbial community, primarily composed of *Firmicutes* and Methanogens, played crucial roles in cellulose degradation and methane production. Notably, *Defluviitoga tunisiensis*, *Syntrophothermus lipocalidus*, and *Pelobacter carbinolicus* were identified as direct metabolizers of cellulose, while *Dehalobacterium* assimilated labelled carbon through cross-feeding. This study provides direct evidence of primary cellulose degraders and sheds light on their genomic composition. By harnessing the potential of lignocellulosic biomass and understanding the microbial communities involved, we can promote sustainable biogas production, contributing to energy security and environmental preservation.

**Supplementary Information:**

The online version contains supplementary material available at 10.1186/s13068-023-02432-x.

## Background

The exponential growth of the world’s population associated with energy consumption have led to a global energy and economic crisis [1]. More than 84% of the world’s total energy demands is supported by non-renewable fossil resources such as coal, oil, and natural gas [2], which are not only limited sources but also have adverse effects on the environment due to the emission of greenhouse gases (GHGs) into the atmosphere.

One of the promising alternatives to fossil-derived energy is the biogas produced through anaerobic digestion (AD) of renewable feedstocks [1]. This is mainly due to the many benefits of AD technology, which has largely been implemented over the last century. The utilization of existing feedstocks for biogas production has the potential to generate energy corresponding to 6–9% of the world’s total energy consumption [3]. Many different biodegradable feedstocks have been utilized for commercial biogas production, such as corn, woodchips, straw, industrial wastewater, animal manure, and many others [2]. Lignocellulosic biomass is the most abundant biomass on earth with more than 180 billion tonnes being produced every year with only 8.2 billion tonnes being currently used [4].

The composition of lignocellulosic biomass depends on its origin, but it primarily consists of cellulose (35–50%), hemicellulose (20–35%), and lignin (10–25%), which categorizes it as a highly recalcitrant biomass structure [5]. Therefore, the hydrolysis step in the AD often becomes the rate-limiting step towards the utilization of lignocellulosic biomass [6].

Lignocellulosic biomass has a tremendous potential to serve as a feedstock for methane production, however, the complexity and the high amounts of solids of the plant biomass are major challenges in digesting this type of biomass. These challenges are caused by the lack of fundamental knowledge on the identity, stress tolerance, and optimal growth conditions of the microbiome involved in this anaerobic utilization of lignocellulosic biomass. Knowledge on understanding the microscopic aspect of lignocellulosic anaerobic digestion is therefore a prerequisite for future controlled and optimized biowaste management and a starting point for fully exploiting second generation energy feedstocks.

High-throughput sequencing methods have provided tools to determine the identity, but also represent a possibility to address functional potential. Despite various process conditions affecting the active microbial community and microbial indicators, the lack of essential knowledge in these areas continues to pose a significant obstacle to the advancement of microbial-based control strategies in, e.g., ADs. Two major problems related to strict metagenomic approaches is that a large part of the microbiome in AD remains unknown and can therefore only be classified on higher taxonomic ranks, and potential functions indicated by metagenomic analysis do not necessarily translate into active processes involved in the substrate conversion [7]. Different studies have identified potential key microbial communities involved in degradation of lignocellulosic biomass and cellulose, but common for most of these studies is that they are based on correlation between degradation rates of lignocellulosic straw and/or cellulose with changes in specific genes or enzymes involved in cellulose degradation [8, 9].

In this study, the main goal was to obtain and characterize the identity and functionality of a long-term adapted anaerobic microbiome able to degrade wheat straw as the sole substrate. Protein stable isotope probing (protein-SIP) was used to reveal the key-players on cellulose degradation by tracking the assimilation of carbon from ^13^C-labelled cellulose into the newly synthetized proteins. Hereby, providing a direct link between identity and function.

## Results

### Performance of the reactor

The reactor reached biogas production stability after 8 operational weeks with straw as the sole carbon and nitrogen source. The volumetric methane production (VMP) and the methane yield (MY) during the last six operational weeks (from week 8 to week 14) were 0.205 ± 0.06 L CH_4_ L^−1^ d^−1^ and 0.201 ± 0.07 L CH_4_ gCOD_rem_^−1^, respectively. This methane yield corresponds to 57% of the theoretical methane yield.

### Phylogenetic microbial community composition of straw-fed semi-continuous reactor

The microbial community composition and stability of the semi-continuous bioreactor was evaluated by amplicon sequencing of the 16S rRNA (microbiome) and *mcrA* (methanogens) genes. For the bacterial population at least 107,266 reads per sample passed filtering and for the methanogenic population at least 25,434 reads per sample passed filtering.

**Fig. 1:**
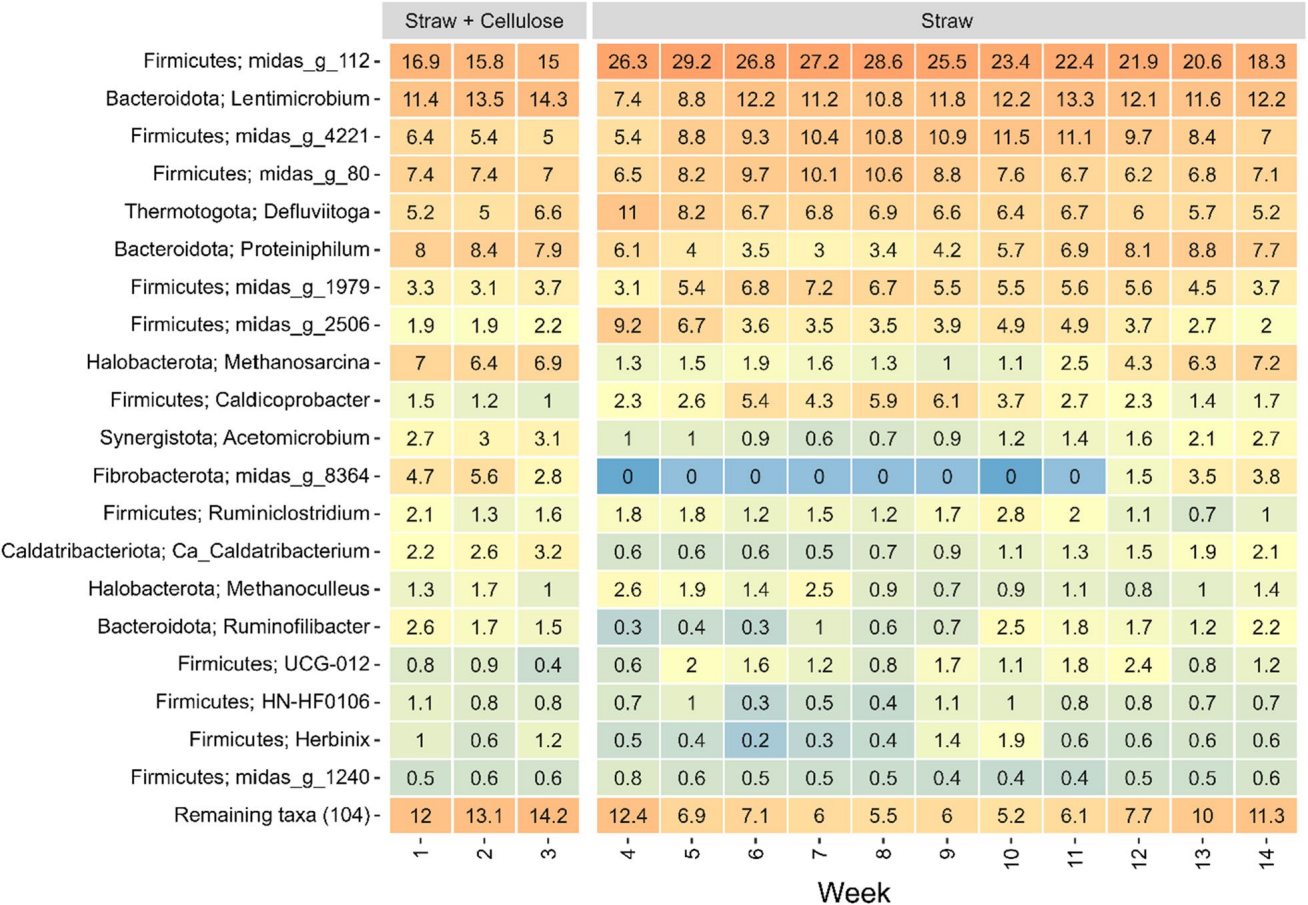
Heatmap of the 20 most abundantly observed microbial populations, displayed at genus level or otherwise best possible taxonomic classification, across the 14 weeks of sampling

**Fig. 2:**
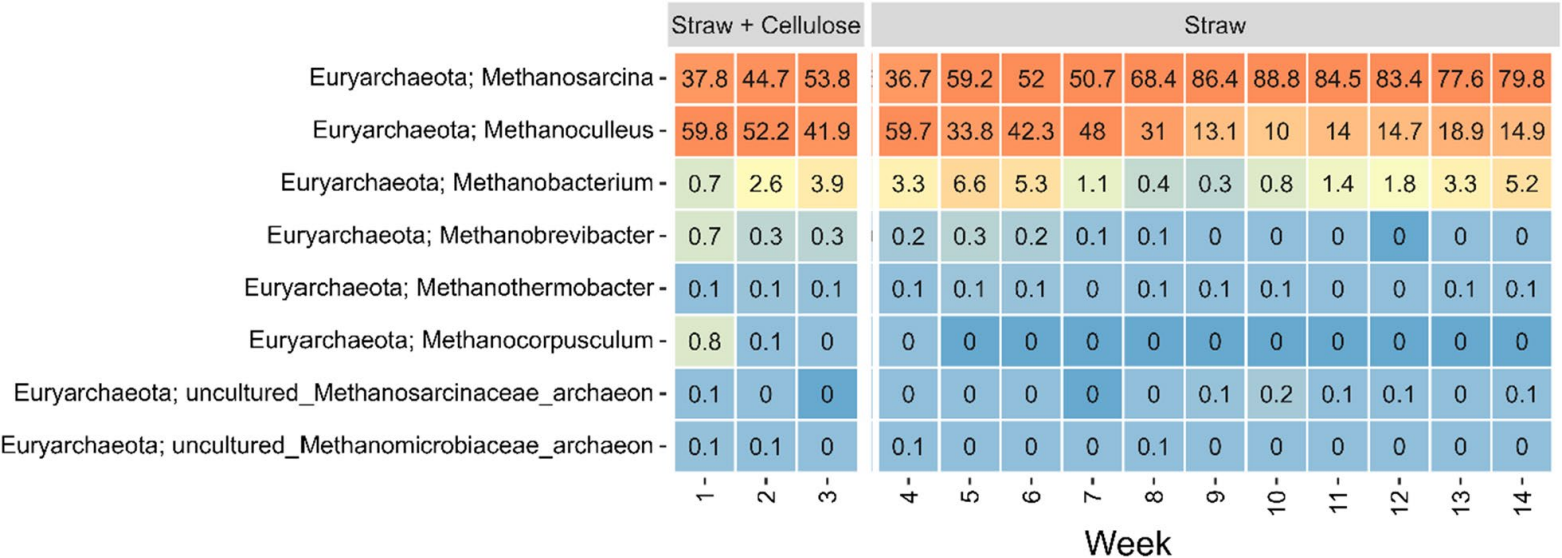
Heatmap of the observed methanogenic populations, displayed at genus level or otherwise best possible taxonomic classification, across the 14 weeks of sampling

The three most abundantly detected genera (based on 16S rRNA gene amplicons) were midas_g_112, *Lentimicrobium*, and midas_g_4221 (Fig. 1). Among the methanogens (based on *mcrA* gene amplicons), the three most abundant genera were *Methanosarcina*, *Methanoculleus*, and *Methanobacterium* (Fig. 2), constituting more than 98% of the methanogens throughout the 14 weeks of sampling.

**Fig. 3:**
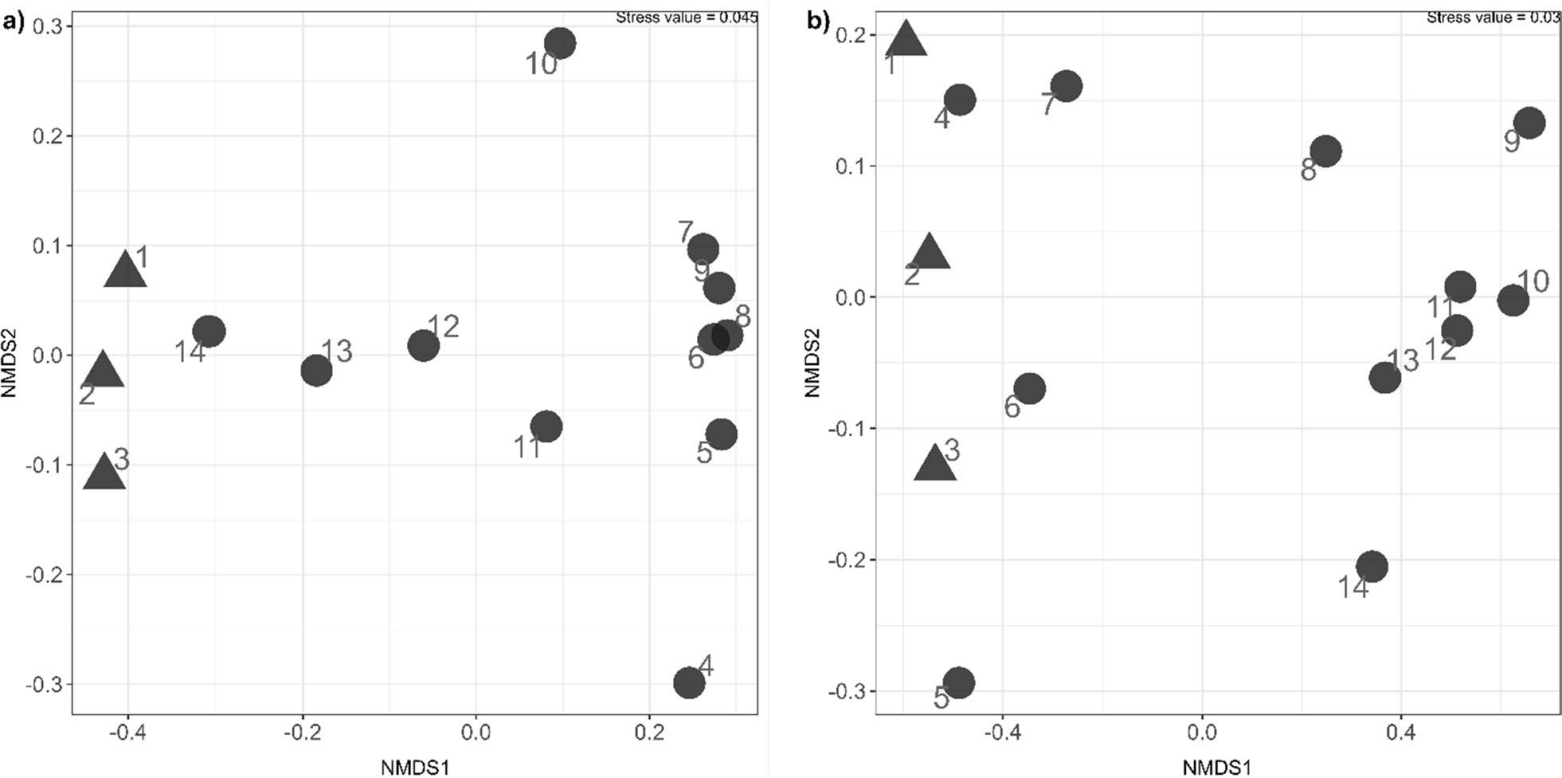
Non-metric multidimensional scaling (NMDS) analysis of differences in **a** the microbial community and **b** the methanogenic community. Numbers next to the points indicate the week of sampling. Black circle indicates the feed being a mix of cellulose and straw and black up-pointing triangle indicates the feed being only straw

The most noticeable change in the microbial community seems to occur right after the feed has been changed to only straw (week 4 and forward). This difference and the evolution of the microbial communities is also illustrated by the non-metric multidimensional scaling (NMDS) (Fig. 3). The two phyla seemed to be affected the most by this change of feed is *Firmicutes* (increased abundance) and *Fibrobacterota* (decreased abundance) (Fig. 1).

A shift in the microbial community was observed immediately after changing the feed to solely consist of straw, starting from week 4 (Figs. 1, 2, and 3). This shift led to a reduction in the relative abundance of various identified organisms, including representatives of the genera *Methanosarcina*, *Lentimicrobium*, *Proteiniphilum*, and *Acetomicrobium*. Notably, the organism with the most significant decrease in relative abundance was the midas_g_8364.

### Metagenome

The metagenome generated from the biomass (corresponding to operational week 14) yielded a grand total of 8,780,000 reads, which when assembled resulted in 411,311,107 bp divided into 10,900 scaffolds with an N50 of 139,335 bp. The metagenome assembly consisted of 89.7% *Bacteria*, 9.8% *Archaea*, and 0.5% *Viruses*, divided across a total of 42 different phyla, with *Firmicutes* being the most abundant representing 31% of all identified scaffolds.

### Proteins identified from the SIP-incubations

Protein analysis of the incubations generated a grand total of 78,705 peptides across all 10 samples from the SIP incubation. Stringent filtering for isotopically altered peptides was applied and the relative isotope abundance (RIA) was set to be equal to or higher than 10%. This criterium yielded a total of 177 labelled peptides (Table S1). All 177 peptides showed significant isotopic labelling profiles, both RIA and manual curation of the individual isotopic distributions verified clear labelling. Already after 4 h of incubation, labelled proteins were identified.

**Fig. 4:**
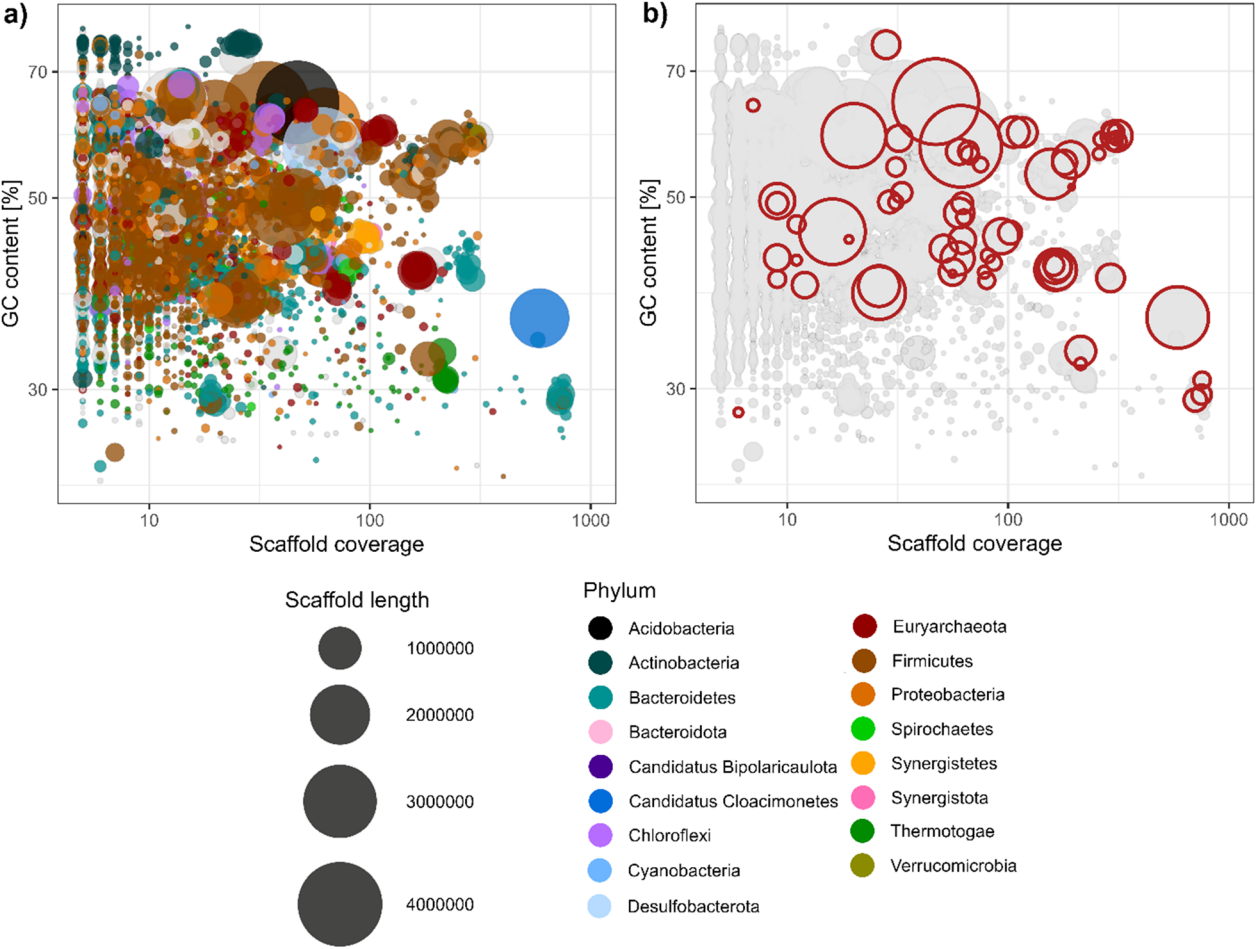
The scaffold coverage and GC content from generated metagenome are plotted. The dot size indicates the scaffold length, and with a minimum length of 5000 bp. **A** Colouring is according to the phylogeny (phylum). **B** Scaffolds where ^13^C-labelled proteins were found are marked

Most of the labelled peptides belonged to organisms representing the phylum *Firmicutes* (Fig. 4), while a small number of the labelled peptides were associated to the phyla *Euryarchaeota*, *Synergistetes*, *Proteobacteria*, *Thermotogae*, and *Bacteroidetes*. Peptides with the highest RIA (> 80%) include representatives of hypothetical proteins, cyclodextrin-binding protein, GTP-sensing transcriptional pleiotropic repressor CodY, proteasome subunit beta, and elongation factor Tu (Additional file 1: Table S1).

**Fig. 5:**
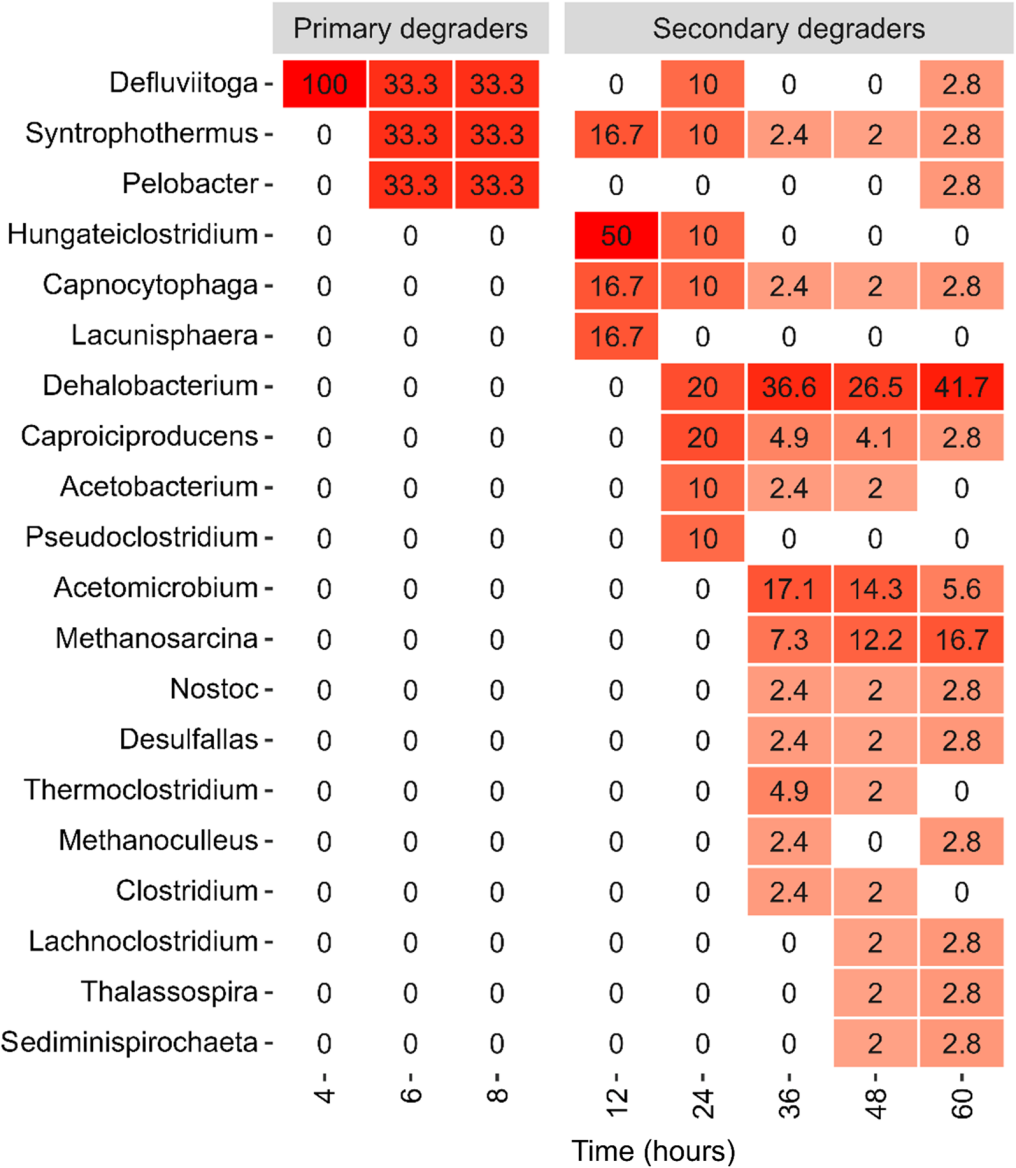
Heatmap of the top 20 organisms assimilating labelled carbon, based on number of labelled proteins identified for each organism to each sampling time (4 h, 6 h, 8 h, 12 h, 24 h, 36 h, 48 h, and 60 h). Colour intensity refers to the scale (0–100). Primary and secondary degraders were predicted based on isotopic profiles (see “Discussion”)

The genera *Defluviitoga* was observed to assimilate the labelled carbon as the primary degrader after just 4 h of incubation, and short after (6 and 8 h of incubation) both *Syntrophothermus* and *Pelobacter* were also identified as having assimilated labelled carbon (Fig. 5). *Dehalobacterium* was observed to be actively incorporating ^13^C from the labelled substrate after 34 h of incubation and throughout the incubation.

### Phylogenetic characterization and metabolic potential of identified cellulose degraders

From the metagenome a total of 25 MAGs were extracted, and hereof 6 high-quality MAGs according to the stringent MIMAG high-quality draft requirements [10] (Table S2). Labelled proteins were affiliated with one of these HQ-MAGs, bin.3, three times throughout the different sampling points. Bin.3 was found to have a completeness of 100%, contamination of 1.72%, 0 ambiguous bases, and is made of one contiguous sequence of 3,538,957 bp with a GC content of 57.3% (Table S2). The HQ-MAG (bin.3) contains 3,659 genomic objects and 51 RNA-coding genes (48 tRNA and 3 rRNA). Furthermore, bin.3 has been taxonomically classified as a *Pelobacter carbinolicus*, using the taxonomic classifier Kaiju [11].

## Discussion

The aim of the study was to identify and elucidate microorganisms actively involved as the primary degraders of lignocellulosic biomass. A reactor was operated to obtain a long-term adapted microbiome to degrade wheat straw under anaerobic and thermophilic conditions, followed by a protein-SIP experiment to further investigate the key-players on the cellulose degradation. Utilizing crop residues, like wheat straw, for anaerobic digestion presents a promising opportunity to capture energy through biogas production while recycling valuable nutrients for agricultural land. By harnessing the untapped potential of these crop residues, we can foster sustainable practices that contribute to energy security and soil health, forging a path towards a greener and more efficient agricultural landscape.

### Reactor performance

The semi-continuous reactor performance for biogas production yielded a VMP and MY of 0.205 ± 0.06 L CH_4_ L^−1^ d^−1^ and 0.201 ± 0.07 L CH_4_ gCOD_rem_^−1^, respectively, which corresponds to 57% of the theoretical methane yield. It is suggested that the composition of the wheat straw has influenced the biogas production due to its recalcitrant nature and effect on the hydrolytic stage of the AD. These results are in agreement with previous studies [12], in which the obtained methane yield ranged from 0.231 to 0.296 L CH_4_ gVS^−1^, corresponding to 65 and 85% of the theoretical methane yield, in similar experimental set-up treating wheat straw at the same OLR. It is suggested that the highest methane yield (85%) obtained by the authors may be a result of co-digesting wheat straw with sewage sludge.

### Changes in the bacterial and methanogenic community

Regarding methanogenesis, *Methanosarcina* is known to have the capability to utilize all three known pathways and a minimum of nine different substrates, allowing it to utilize all three products from acetogenesis. Moreover, this genus has been reported to exhibit exceptional tolerance to high ammonium concentrations, high salt concentrations, changes in pH, and temperature [13]. The presence of high diversity in a community is generally considered a sign of ecosystem stability, as it provides a mechanism for coping with stress and environmental changes. This flexibility allows the sensitive population to adapt robustly and alter the preferred pathway of methanogenesis if needed.

In contrast, *Lentimicrobium* and *Acetomicrobium* possess chemoorganotrophic metabolism and act as fermenters of a narrow range of carbohydrates [14–17]. As for *Proteiniphilum*, it has been shown that substrates such as starch and sugars can support anaerobic growth [18]. In our study, we observed the presence of *Proteiniphilum* throughout the 14 weeks, with relative abundances reaching up to 8.8% of the total population (Fig. 1).

A few organisms, including *Defluviitoga* and *Caldicoprobacter*, were observed to increase in abundance right after the change in substrate from week 3 to week 4. Bacteria belonging to the genus *Defluviitoga* has previously been demonstrated to have a fermentative metabolism in similar environments [19, 20]. Species of this genus can utilize a large variety of carbohydrates, as well as several complex polysaccharides such as chitin and cellulose [21]. Members of *Caldicoprobacter* are chemoorganotrophs, grow under strictly anaerobic conditions, and have a fermentative metabolism with an affinity for sugar substrates [22, 23].

It seems that for the organisms which increased in the relative abundance after change in the substrate (from mix of cellulose and wheat straw till only wheat straw) all prefer sugars/sugar rich substrates, whereas the organisms with decreased abundance resemble organisms with ability to grow on less diverse mixtures of mono- and disaccharides. But common for all these organisms, whether they increased or decreased in abundance, is that after 8 weeks of adaptation to the straw substrate the microbial community is almost back at the starting point (Figs. 1 and 3). So, the shift in community composition appeared to be temporary and returned to the original after 14 weeks.

### Protein-SIP analysis

Several peptides with a clear isotopical shift were found. Some of these labelled proteins were identified as hypothetical proteins (103 unique accessions), flagellin (18 unique accessions), ABC transporters (5 unique accessions), and elongation factor Tu (5 unique accessions). All of the above-mentioned proteins have common features of high turnover rate, highly abundant among many different organisms, and the detection of multiple of these proteins are in line with the results of previous studies where protein-SIP was used in different settings of AD [24–27]. To ensure a high significance in the detection of labelled proteins, we have in this study applied very conservative restrictions (RIA > 10%) and both automatically and manually curated the data.

The isotopic distribution of most of the identified peptides followed a tailed distribution, revealing that the organisms synthesizing the peptides got the labelled carbon through cross-feeding [28, 29] and not through direct metabolization of the labelled substrate. This was similarly observed for the two methanogens (*Methanosarcina* and *Methanoculleus*). When organisms directly metabolize isotopically labelled substrates, the resulting isotope pattern closely resembles a normal distribution [28, 29]. However, when cross-feeding occurs, meaning the usage of labelled intermediates from degradation processes that accumulate in the culture, it leads to tailed distributions (negative skewness). *Dehalobacterium formicoacenticum* was seen to assimilate labelled carbon at the end of the incubation (after minimum 12 h). This organism has previously been reported not to be able to metabolize sugars for energy [30, 31]. Therefore, the labelled carbon assimilation must derive from a cross-feeding of labelled intermediates from the degradations process of cellulose, which is also supported by the isotopic distribution.

Three organisms have been identified as direct metabolizers of the labelled cellulose: *Defluviitoga tunisiensis*, *Syntrophothermus lipocalidus* and *Pelobacter carbinolicus*. *Defluviitoga tunisiensis* was the first to assimilate the labelled carbon from the cellulose. This organism has previously been reported as a cellulose degrader with a high H_2_-production ability [32]. The organism *Syntrophothermus lipocalidus* has not to our knowledge previously been described as able to degrade/metabolize cellulose. However, it has previously been shown that 3.4% of all genes in this organism were affiliated to carbohydrate transport and metabolism [33]. It was a bit surprising to see *Pelobacter carbinolicus* identified as one of the main degraders of the cellulose, considering that an inability to ferment sugars through glycolysis is thought to be a defining characteristic for this species [34]. However, looking into the genome of *Pelobacter carbinolicus* it was discovered that it encodes for proteins involved in sugar uptake [35]. This study presents clear evidence that this organism is one of the primary metabolizers and assimilates the cellulose. Also, from the genomic material of the HQ-MAG (bin.3), classified as a *Pelobacter carbinolicus*, multiple genes encoding carbohydrate degradation is observed, supporting that this organism can take up and metabolize sugars, such as cellulose.

This study offers a significant advancement in the construction of a microbiome for biofuel production from recalcitrant lignocellulosic biomass, such as wheat straw, with broader implications for other challenging feedstocks. By employing a combination of state-of-the-art genomic techniques and long-term adaptation strategies, the research identified key microbial species directly involved in cellulose degradation and methane production. This knowledge is valuable as a tool for monitoring and optimizing microbiomes for biofuel production. Instead of relying solely on metagenomic analysis, which provides limited functional insights, the study directly links microbial identity with their functional roles in cellulose degradation. This direct evidence allows for more precise manipulation and engineering of microbial communities, ultimately leading to improved biofuel production efficiency and sustainability. Moreover, the findings shed light on the potential of underutilized lignocellulosic biomass resources, contributing to a more environmentally friendly and economically viable bioenergy sector.

## Conclusion

Protein-SIP analysis revealed 3 organisms, *Defluviitoga tunisiensis*, *Syntrophothermus lipocalidus*, and *Pelobacter carbinolicus*, directly metabolizing the cellulose in a digester fed with wheat straw. However, at the end of the incubation (≥ 36 h) *Dehalobacterium* had assimilated labelled carbon, indicating that this organism assimilated the labelled carbon through cross-feeding. This was further validated through the isotopic distribution profiles. This study presents, as one of the first, direct evidence of who are the primary degraders of cellulose by tracking the ^13^C assimilation, while most other studies are based on sequence homology and therefore are limited to identify the organisms with potential to degrade the substrate. In this study, we elucidated organisms involved in the degradation of cellulose, both primary and secondary degraders, and their genomic composition. The results from this study therefore contribute to a better understanding and treatment of lignocellulosic biomass.

## Methods

### Bioreactor description and operational conditions

A semi-continuous stirred-tank bench-scale reactor was operated for 98 days at a hydraulic retention time (HRT) of 30 days in anaerobic conditions. The reactor was made of acrylic with a working volume of 3.5 L, hieght of 31 cm, and internal diameter of 12 cm. The inoculum used was anaerobic sludge collected from a full-scale reactor operated at 50 ℃ treating a mixture of swine and cattle manure, maize silage, and deep litter. The reactor was operated at thermophilic conditions at 55 ℃ and stirred using two horizontal paddles (2 × 8 cm) at 180 rpm. The pH of the reactor was adjusted, when necessary, with NaHCO_3_ and kept at 7 to 7.5.

The reactor was fed manually every second day and operated at an organic loading rate (OLR) of 2 kg m^−3^ d^−1^. The wheat straw was pre-treated mechanically, as previously described [36], and powdered to a particle size of 0.13 mm and it has the following composition (%): total carbon (45.4), total nitrogen (0.62), total solids (97.5), volatile solids (91), cellulose (36.7), hemicellulose (25.2), lignin (23), mineral content (6.7) and other components. Due to the high lignin content of the feed, the reactor was fed with a mixture of cellulose and straw during the first two operational weeks, in which the cellulose content in the feed decreased gradually from 95 to 0%. From the third week on, the only carbon source was the mechanically treated wheat straw. To prevent nutrients shortage, a macro and a micronutrients solution was also added to the feed as described elsewhere [37].

### Community profiling using 16S rRNA and mcrA gene amplicon sequencing

Biomass was collected once a week for 14 weeks from the beginning of the experiment and stored at − 20 ℃ until analysis.

DNA extraction of the sludge was performed using FastDNA Spin Kit for Soil (MP Biomedicals, Denmark), following the manufacturer’s recommendations. The quality of the extracted DNA was evaluated using a TapeStation 2200 and genomic DNA ScreenTapes (Agilent, USA). DNA concentration was determined using Quant-iT HS DNA Assay kit (Thermo Fisher Scientific, USA). Amplicon sequencing variant (ASV) analysis of the V4 variable gene region of the 16S rRNA and the methyl coenzyme-M reductase (*mcrA*) gene were performed as previously described [27] using Illumina MiSeq sequencing platform. Data were analysed using R v4.2.1 [38], RStudio software [39], and visualized using ampvis2 [40] and ggplot2 [41].

### Protein-SIP batch incubations

Labelling experiments were conducted in batch reactors set up in 100 mL serum bottles with 50 mL of working volume incubated with ^13^C-cellulose (IsoLife, Wageningen, the Netherlands) as the sole carbon source. The batch reactors were inoculated with suspended biomass collected from the semi-continuous bioreactor at the final operational day at an F/M ratio of 0.3 and the pH of the bulk was adjusted to 7.8. The batch incubations were kept at 55 °C and constant shaking at 120 rpm on a rotary table. The reactors were sealed with thick butyl rubber stoppers and aluminium crimps and headspace was purged with nitrogen gas (N_2_) for 2 min to ensure anaerobic conditions. The biomass was sampled after 0, 2, 4, 6, 8, 12, 24, 36, 48, and 60 h for protein extraction. All handling and sampling were conducted using strict anaerobic techniques.

### Metagenome and bioinformatics

Biomass from the continuous reactor was used to prepare a reference metagenome. Total DNA was extracted using FastDNA Spin Kit for Soil, following manufacturer’s recommendations (MP Biomedicals, Denmark). For the non-size selected Oxford Nanopore Technologies (ONT) library 1 µg of gDNA was used to create the ONT library. DNA repair, end preparation, adapter ligation, cleanup, and priming were done following the manufacturer’s protocol (MinION; Oxford Nanopore Technologies). In brief, DNA ends were repaired and dA tailed using the NEBNext end repair/dA-tailing module. Sequencing adapters were ligated onto the prepared ends following DNA end repair. The resulting library was loaded onto a single MinION R9.4.1 flow cell and sequenced for 72 h.

Base calling and sequence quality analysis were performed using Guppy v6.0.1 (https://community.nanoporetech.com). Sequence quality was assessed using NanoPlot v1.24.0 [42], and hereafter filtered for quality (> Q9) and length (> 500 bp) using NanoFilt v3.8.6 [42]. Assembly of the reads were done using metaFlye v10.2.0 [43], and the following polish was done using minimap2 v2.17 [44], racon v1.3.3 [45], and medaka v1.0.1 [46]. Prediction of open reading frames in the metagenome, identification of essential genes, and classification the contigs taxonomy were carried out using a script downloaded from https://github.com/Kirk3gaard/misc_scripts/tree/master/prepare_data_for_mmgenome2 was used to prepare the data for analysis in R using the mmgenome2 package (https://kasperskytte.github.io/mmgenome2/).

Metagenome assembled genomes (MAG) were extracted with the MetaBAT v2.12.1 [47] and checked for completeness and contamination using CheckM [48]. Phylogenetic analysis was performed using the FastTree software [49], and a phylogenetic tree was calculated based on the full-length 16S rRNA gene sequences retrieved from the NCBI database [50], using the inferred approximately maximum-likelihood method and a 1000-replicate bootstrap analysis.

### Protein extraction and protein-SIP analysis

Protein extraction was performed as previously described [51] using TEAB (0.05 M TEAB buffer stock, 1.0 mg L^−1^ NaDOC, pH ≤ 8) as resuspension buffer. In-gel digestion of extracted proteins was conducted as previously described [24]. The following desalting and analysis of tryptic peptides by automated liquid chromatograph–electrospray ionization tandem mass spectrometry (LC–ESI–MS/MS) were performed as previously described [52].

The proteomic data were analysed using the MetaProSIP tool [53], in an OpenMS pipeline (https://www.openms.de), using standard settings, as described previously [24, 25], and afterwards manually checked/curated for shape of isotope pattern. The metaproteome, generated from the metagenome and annotated using Prokka (v.1.14) [54], was used as the search database.

### Supplementary Information

**Additional file 1: Table S1**. List of all peptides identified with incorporation of ^13^C. **Table S2**. Assessment of the quality of MAGs recovered from the metagenome.

## Data Availability

All amplicon and metagenome data are available at the European Nucleotide Archive (ENA) under project accession number PRJEB55850. The mass spectrometry proteomics data have been deposited at ProteomeXchange Consortium via the PRIDE [55] partner repository with the data set identifier PXD036712.
